# Dynamics of endophytic fungi composition in *paris polyphylla var. chinensis (franch.) hara* seeds during storage and growth, and responses of seedlings to phytohormones

**DOI:** 10.3389/fmicb.2025.1540651

**Published:** 2025-02-26

**Authors:** Tong Peng, Tao Yang, Jie Sha, Jiang Zhao, Jianwu Shi

**Affiliations:** ^1^Basic Medical Research Centre, School of Medicine, Nantong University, Nantong, Jiangsu, China; ^2^Key Laboratory of Microbial Resources Exploitation and Application, Institute of Biology, Gansu Academy of Sciences, Lanzhou, Gansu, China; ^3^College of Pharmacy, Gansu University of Chinese Medicine, Gansu Academy of Sciences, Lanzhou, Gansu, China

**Keywords:** *Paris polyphylla var. chinensis (Franch.) Hara*, endophytic fungi, phytohormones, phenotypic, antioxidant system

## Abstract

**Introduction:**

Endophytic fungi exhibit diverse interactions with plants, from pathogenic to mutualistic symbiosis, and the community composition is regulated by phytohormones. Yet, the composition and dynamics of endophytic fungi in *Paris polyphylla var. chinensis (Franch.) Hara* (PPC) during fresh seed (FD), sand-stored seed (SSD), and seedling (SS) stages remain unclear. Similarly, the overall impact of phytohormones on the management of endophytic fungal communities is yet to be elucidated.

**Methods:**

We carried out a pot experiment to examine the effects of various stages of PPC seeds and the external addition of three phytohormones, namely, melatonin (MT), strigolactone (SL), and 24-epibrassinolide (BR) on the endophytic fungi of PPC seedlings. This was done through internal transcribed spacer (ITS) amplicon sequencing.

**Results:**

The study of the endophytic fungal microbiome in FD, SSD, and SS stages of PPC revealed an increased richness and diversity of fungi during the SS stage, with significant changes in community composition observed. We found that *Sordariomycetes* played a crucial role in this process, potentially contributing to the establishment and growth of PPC seedlings. Additionally, this study investigated the influence of phytohormones on the phenotypic and physiological characteristics of PPC and its endophytic fungal community. Our results demonstrated that MT and SL significantly increased PPC biomass by 69.32 and 15.23%, respectively, while 2 mg/L of BR hindered the growth of PPC roots. MT, SL, and BR not only induced significant changes in the composition and diversity of the endophytic fungal community in PPC but also affected biomass potentially through specific regulation of potential biomarkers. Furthermore, phytohormones were shown to indirectly modify the endophytic fungal community by altering antioxidant system in plants.

**Conclusion:**

This study provides novel insights into the dynamic changes of microbial communities in the FD, SSD, and SS stages. Furthermore, the differences among various phytohormones ultimately enhance our predictive understanding of how to directly or indirectly manipulate the plant microbiome to improve plant health.

## 1 Introduction

*Paris polyphylla var. chinensis (Franch.) Hara* (PPC) is a perennial flowering herb that belongs to the *Melanthiaceae* family. Formerly, it was classified under the *Liliaceae* family and is endemic to China. This species is listed in the International Union for Conservation of Nature’s Red List (IUCN) (Wang et al., 2019). Additionally, the 2020 edition of Chinese Pharmacopeia includes PPC as one of the two original medicinal plants utilized in Paridis Rhizoma (State Pharmacopoeia Commission of the Prc, 2020). Modern pharmacological studies have demonstrated that the primary active component, steroid saponins, exhibits significant biological activities such as anti-tumor, anti-inflammatory, and hemostatic properties. Its efficacy and scope of application continue to expand (Guan et al., 2024). Research on the Chinese pharmaceutical market indicates that PPC is a key ingredient in over 80 traditional Chinese medicine formulations, with annual sales ranging from 800 to 1,050 tons. The annual consumption far exceeds its natural growth capacity, posing a threat of depletion to its wild medicinal resources (Cunningham et al., 2018).

Endophytic microorganisms in plants primarily originate from two sources: the external environment on the plant surface, and seeds (Liu et al., 2020). Endophytic microorganisms, including bacteria, fungi, and actinomycetes, are transmitted vertically between successive plant generations (Shade et al., 2017). The relationship between endophytic fungi and plants is extensive, ranging from being potential pathogens or saprophytes to mutualistic symbiosis (Zhang et al., 2024). Consequently, this intergenerational transmission significantly impacts plant health, quality, productivity, and microecology (Truyens et al., 2015; Nelson, 2018). In agricultural production, due to the lack of cost-effective tissue culture techniques, PPC can only be propagated via seeds. Fresh PPC seeds are immature, both morphologically and physiologically. Following 80 days of warm wet sand stratification before sowing, the PPC seeds complete their entire process of embryo morphological maturation, from nearly spherical to elliptical to cylindrical embryos. After an additional 60 days of low-temperature stratification, the seeds reach physiological maturation. During the sand stratification process, the seeds may be invaded and colonized by external fungi (Zhang et al., 2023). After reaching morphological and physiological maturity, changes in the internal nutritional and hormonal physiological levels of seeds alter the habitat and promote the growth of certain microorganisms within the seeds (Okunishi et al., 2005). Following seed germination, the seed coat gradually sheds, triggering the reassembly of microbial communities on the plant surface and within its tissues. These communities progressively converge toward a similar composition, with the presence of endophytic fungi serving as a key indicator of the completion of this assembly process (Shahzad et al., 2018). However, compared to the seed stage, this phase typically undergoes significant compositional changes. Studies have shown that the endophytic bacterial communities in seeds vary not only due to the seed development process and environmental conditions (Zhang et al., 2023), but also exhibit significant differences among different plant species (Links et al., 2014). Due to their positional advantage, endophytic fungi within the seeds may influence plant growth and adaptability, from seeds to seedling emergence, and continue to affect plant development over time. Therefore, understanding the dynamics of fungi during the processes of fresh seeds, seed storage, and early seedling germination is crucial for the selection and maintenance of endogenous microorganisms beneficial to plant growth and health, as well as monitoring changes in pathogenic bacteria.

The radicle begins to develop after seed germination, leading to seedling growth. PPC typically grows in valleys, beneath forests, or amid shrubbery, where plants compete for sunlight, air, and water. Relative to the fragility of seedlings during the early stages of growth, their swift and robust development influences subsequent growth and yield (Carrera-Castaño et al., 2020). Therefore, the early growth of seedlings is crucial because it enhances the plant’s ability to compete with other species in the field. Phytohormones have long been used to address sluggish early growth of plant seedlings. Research shows that phytohormones play a broad role in plant physiological processes, regulating plant growth and development, and they are critical in shaping the plant microbiome (Eichmann et al., 2021). These hormones can act as nutrients and signaling molecules to regulate the growth and metabolism of endophytic microorganisms (Xu et al., 2018), or selectively restrict the growth of certain members within the plant microbiome by modulating the reactive oxygen species (ROS) system (Stringlis and Pieterse, 2021). Brassinosteroids (BR) are a type of efficient, broad-spectrum steroidal plant hormone involved in processes like cell elongation, tissue differentiation, and responses to abiotic stress (Talaat and Shawky, 2012). Research confirms that BRs promote plant growth by enhancing antioxidant capacity and photosynthetic efficiency (Sytar et al., 2019). Moreover, BRs reportedly increase rhizosphere fungal diversity (Song et al., 2023), and positively impact arbuscular mycorrhizal (AM) symbiosis (Ren et al., 2021). Strigolactone (SL), a novel carotenoid-derived phytohormones is extensively involved in plant growth, including seed germination, seedling development, bud branching, stem elongation, and lateral root formation (Al-Babili and Bouwmeester, 2015). In terms of interactions with microorganisms, SL, similar to BR, has also been reported to participate in AM symbiosis. It reportedly influences significant differences in fungal community composition, but without significant differences in bacterial community composition (Carvalhais et al., 2019). SL can enhance plant resistance to pathogenic fungi (Marzec, 2016). Melatonin (N-acetyl-5-methoxytryptamine) is a natural indoleamine compound that aids in ROS scavenging, photosynthetic system regulation, leaf senescence delay, and tolerance promotion to some biotic stresses by reducing the abundance of several harmful fungi in the rhizosphere soil (Back, 2021; He et al., 2023). While previous studies successfully manipulated specific endophytic fungi in plants through the exogenous application of synthetic plant hormones, there have been no reports on manipulating endophytic fungi at the overall community level using phytohormones.

In this study, we delineated the endophytic fungal communities in PPC throughout three stages: fresh seeds (FD), sand-stored seed (SSD), and seedling (SS), and in response to the treatment of three phytohormones (MT, SL, and BR). The study objectives were as follows: (1) to examine the dynamic changes in endophytic fungi within PPC seeds from the fresh to the stored and seedling stages; (2) to scrutinize the specific impacts of the exogenous addition of three phytohormones on these endophytic fungi; and (3) to further analyze the correlation between the PPC endophytic fungal microbiota and certain physiological parameters, to understand the influence of these phytohormones on the assembly of PPC endophytic fungal microbiota.

## 2 Materials and methods

### 2.1 Site description

The study was carried out at Biological Research Institute of Gansu Academy of Sciences and Heping testing base, Longnan (36.008 N, 103.970 E), Gansu, China. The experimental site is in the eastern monsoon region, and has a cold, semi-humid, and rainy climate, with an altitude of 1,700 m. The growing season of PPC seedlings is from December 2018 to October 2019. The soil is classified as Calcaric Cambisol according to the Food and Agricultural Organization (FAO) classification system. The initial physicochemical characteristics of the soil were: pH = 7.84, organic matter = 1.19 g/kg, TN = 0.63 g/kg and available K = 3.42 mg/kg. During the process of collecting the soil, large particles of impurities, including plant litter, plant roots, and gravel, were removed. The soil was thoroughly mixed before being filled into the flower pots.

### 2.2 Plant materials and experimental design

**FIGURE 1:**
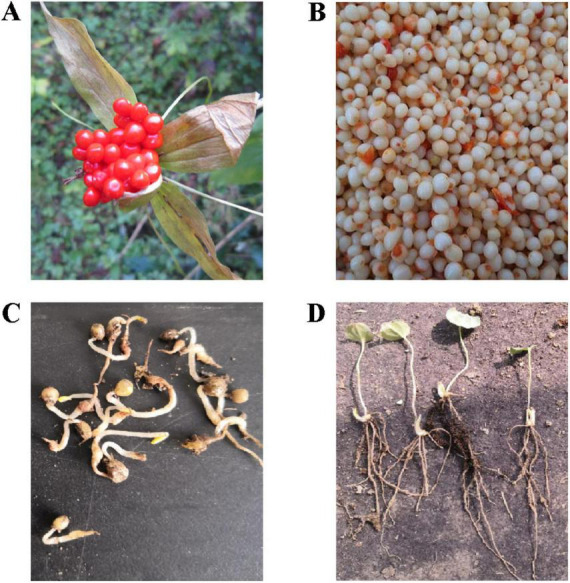
Experimental sampling. **(A)** Fresh fruit samples. **(B)** Fresh seed samples. **(C)** Post-stratification seed samples. **(D**) Seedling stage samples.

In this study, PPC samples were collected at three stages: fresh seeds (FD), sand-stored seeds (SSD), and the seedling (SS) stage. The fresh fruits (Figure 1A) were collected from Guanshan, Huating City, Gansu Province, China, at an elevation of 2,000 m (35°200 N, 106°396 E). To obtain fresh seeds, the fruits were carefully processed by removing the pulp and peel (Figure 1B). Before sowing, fresh seeds require a two-step temperature-alternating sand storage protocol to break dormancy. Initially, seeds undergo a warm stratification phase of approximately 40 days at 18°C, during which PPC seeds complete the full process of embryonic morphological maturation, transitioning from nearly spherical to elliptical, and finally to a cylindrical embryo. After this, seeds are subjected to a cold stratification period of 60 days at 4°C, at which point the seeds attain physiological maturity. Seeds collected at this stage are used as the post-stratification sample (Figure 1C). The sand-stored seeds are then transplanted into plastic pots (17.6 cm × 26.5 cm × 23 cm). The pre-germination treatment of seeds is conducted under moist and cool conditions (with a 70% shade net) to ensure rapid and uniform germination following sowing. Post-germination, the seedlings are grown under natural temperature conditions, and seedling roots at the thirteenth week are collected as samples for the seedling stage (Figure 1D).

The present study implemented four treatments, which comprised individual inoculations with MT (MT), SL (SL), BR (BR), and an equivalent volume of sterile distilled water as the control (CK). The treatments were arranged in a randomized block design with three replicates per treatment, containing three seedlings per pot. The concentrations of MT, SL, and BR used in the experiment were in line with previous research by Ye et al. (2022), all at 2 mg/mL and 15 mL per pot. Starting from the 8th week of the seedling stage, applications were made once a week for five consecutive weeks. Seedling roots were collected in the 13th week to be used as samples for the seedling stage evaluation.

All collected samples underwent surface sterilization. Initially, the samples were rinsed with sterile distilled water at least three times, or until no turbidity was observed. Subsequently, the samples were immersed in a solution of 5% NaClO for 10 min, followed by three additional rinses with sterile distilled water. After this, 150 mL of the final rinse water was plated onto potato dextrose agar plates. The plates were then incubated at 28°C for 72 h to confirm the effectiveness of the seed surface sterilization. The samples were promptly stored at −80°C for subsequent utilization. The experiment included six treatments, named FD, SSD, SS (CK), MT, GR, and BR. Each treatment had three replicates, resulting in a total of 18 samples.

### 2.3 Measurement of phenotypic, physiological, and hormone content of PPC

After phytohormone treatment, we randomly selected three pots per treatment group to measure root length, shoot length, and root fresh weight. Plant samples were placed in an oven at 105°C for 30 min to inactivate the enzymes. Next, they were dried at 70°C until they reached a constant weight; we then recorded the biomass. We calculated all indicators based on an average of three plants per pot. We transferred 1.0 g of mixed plant samples to a plastic centrifuge tube containing 18 mL of precooled 10 mM phosphate-buffered saline (130 mM NaCl, 7 mM Na_2_HPO_4_, and 3 mM NaH_2_PO_4_; pH 7.4). The plants were then homogenized using a homogenizer. Afterward, we centrifuged the homogenized sample at 3,000 rpm/min for 20 min at 4°C. We then collected and stored the supernatant at 4°C for physiological index measurement. In this study, we determined four physiological indices: malondialdehyde (MDA) content, hydrogen peroxide (H_2_O_2_), superoxide dismutase (SOD) activity, and catalase (CAT) activity. We measured all indices using plant Enzyme-Linked Immunosorbent Assay (ELISA) kits (Mibio, Shanghai, China) following the kit instructions. We purchased test kits for MDA (Lot. No.20200521), H_2_O_2_ (Lot. No.20200526), SOD (Lot. No.20200619), and CAT (Lot. No.20200603) from Suzhou Keming Biotechnology Co., Ltd. Net photosynthetic rate (Pn), stomatal conductance (Gs), inter-cellular CO_2_ (Ci), and transpiration rate (Tr) were determined by a portable infra-red gas analyzer—photosynthesis system, LI-6400XT (Lincoln, NE, United States) between 10 a.m. and 12 noon. The contents of Salicylic acid (SA), MT, SL, and BR in plant were measured using plant ELISA kits (Mibio, Shanghai, China) according to the provided instructions.

### 2.4 Soil microbial DNA extraction and sequencing

The total DNA of each sample was extracted using a DNeasy PowerSoil DNA Extraction Kit, following the manufacturer’s instructions (QIAGEN). The identification region for the analysis of fungal diversity, ITS I variable regions were amplified with universal primers (primers: ITS1F 5′-CTTGGTCATTTAGAGGAAGTAA-3′; ITS2 5′-GCTGCGTTCTTCATCGATGC-3′). Sequencing was conducted on an Illumina NovaSeq 6,000 instrument post PCR amplification and purification. Sequencing reads were processed to obtain effective sequences using barcodes for all samples. Then, alignment, filtration, and removal of chimeras from reads were accomplished using software applications such as Pandaseq, Prinseq, and Usearch. Sequences were clustered using Usearch at a similarity threshold of 0.97, chimeras were filtered from the resultant cluster sequences, and OTUs (operational taxonomic units) were collected for species classification. Then, sequences with a similarity threshold of 0.97 were aligned against the representative sequences of the OTU for additional analysis. Taxonomic data annotation was done for representative sequences of fungi using UNITE (version 8.0)^1^ databases, respectively. The microbial community structure was investigated using the Illumina NovaSeq 6,000 platform (Nanjing GENEPIONEER Biotechnology Co., Ltd., China) for high-throughput sequencing of fungal ITS rRNA.

### 2.5 Statistical analysis

Alpha diversity indices, including community richness (Chao1) and community diversity (Shannon), were utilized to indicate dissimilarity among samples. The beta diversities across the different stages were determined by principal coordinates analysis (PCoA), and the significant differences were determined by permutational multivariate analysis of variance (PERMANOVA). Non-metric multidimensional scaling (NMDS) and multivariate PERMANOVA were employed to reveal the succession of endophytic fungi, as well as to measure differences in community structure among different treatments. Co-occurrence network analysis and visualization were completed using Gephi software (v.0.9.2), and only significant relationships were included (R > 0.85, *p* < 0.05). Four node-level topological features were calculated per node: degree, betweenness, closeness, and eigenvector centrality. To investigate relationships between phytohormone contents, physiological properties, and endophytic fungal communities on PPC biomass, partial least squares path modeling (PLS-PM) was performed using the “plspm” package in R.3.1.3. Random forest analysis was performed using the “randomForest” package in R.3.1.3. Significant pearson correlation (*p* < 0.05) between phytohormone and microbial taxa were analyzed and visualized using the interactive networks in R.3.1.3, Cytoscape 3.3.0. Only significant relationships were included (R > 0.5, *p* < 0.05) in co-occurrence network analysis.

The data was analyzed using Analysis of Variance (ANOVA) in SPSS 17.0 for Windows (IBM SPSS Inc., Chicago, United States), and the results are expressed as the mean ± standard deviation. A *p*-value less than 0.05 suggested significant differences, while a *p*-value less than 0.01 indicated extremely significant differences.

## 3 Results

### 3.1 Diversity and endophytic fungi communities composition of different growth stages

**FIGURE 2:**
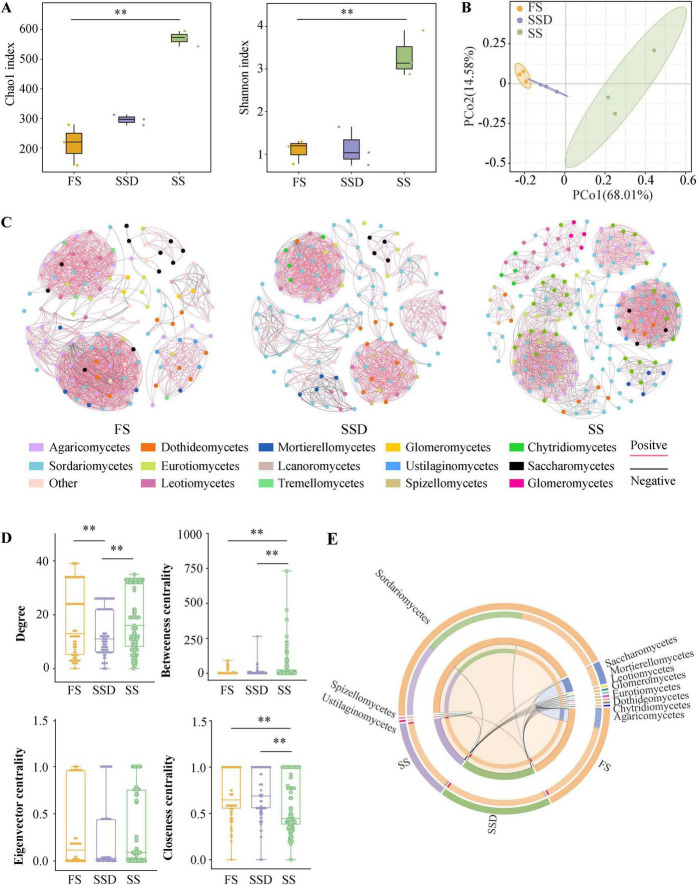
Endophytic fungal diversity and community composition in *Paris polyphylla var. chinensis (Franch.) Hara* (PPC) at fresh seed (FD), sand-stored seed (SSD), and seedling (SS) stages. **(A)** Alpha diversity data (Chao1 and Shannon index). **(B)** β-diversity data [Principal co-ordinates analysis (PCoA) based on Bray-Curtis dissimilarities]. **(C)** Co-occurrence networks at class level based on a correlation analysis. A link represents a significant correlation (Spearman’s |*r*| > 0.85 and FDR-corrected *p* < 0.05). Red and black links represent positive and negative relationships, respectively. **(D**) Comparison of node-level topological characteristics at class level. **(E)** Composition of the endophytic fungal communities at phylum level. **p < 0.01 (Dunn’s test).

In this study, the ITS gene was sequenced to identify endophytic fungi at three key growth stages of PPC: FS, SSD, and SS. A total of 831,954 high-quality sequences were generated from nine samples. These were classified into 209 fungal OTUs at a 97% clustering level, with 131, 141, and 185 OTUs in FS, SSD, and SS, respectively. Alpha diversity showed that the fungal richness (Chao1) and diversity (Shannon) during the SS stage were significantly higher than those of FS and SSD, with no significant difference between FS and SSD (Figure 2A). Furthermore, the PCoA, based on the Bray-Curtis metric (Figure 2B), revealed substantial differences in fungal composition between SS and the other two stages. Specifically, FS and SSD samples partially overlapped after clustering, while SS samples were separate from the other two stages on the first coordinate axis. The overall network of endophytic fungal communities at different stages portrayed a clear symbiotic pattern. The fungal communities of FS, SSD, and SS had meta-network node numbers of 130, 142, and 185, respectively, with significant edge numbers of 949, 951, and 1,700, respectively (*p < 0.05*) (Figure 2C). Generally, almost all links between OTUs exhibited positive correlations. Further comparison of the specific node topological characteristics showed that the betweenness centrality and closeness centrality values of SS were notably greater than those of FS and SSD (Figure 2D). Overall, FS and SSD endophytic fungi were significantly different from the SS stage. The dominant phyla of endophytic fungal communities under different treatments were *Sordariomycetes* (79.048%), *Saccharomycetes* (5.948%), *Leotiomycetes* (0.118%), *Eurotiomycetes* (0.116%), *Mortierellomycetes* (0.091%), *Dothideomycetes* (0.024%), *Agaricomycetes* (0.017%), *Chytridiomycetes* (0.002%), *Glomeromycetes* (0.002%), *Spizellomycetes*, and *Ustilaginomycetes* (Figure 2E).

### 3.2 Discrimination of endophytic fungi at different stages of PPC seeds

**FIGURE 3:**
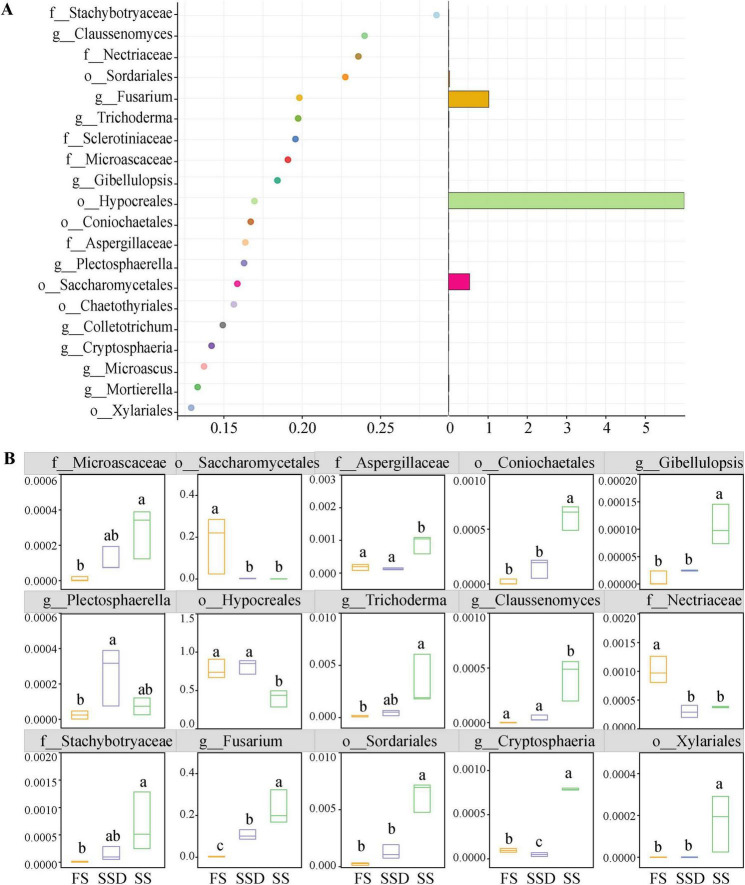
Predictor importance of the top 20 endophytic fungi taxonomic biomarkers (detected by random forest model) **(A)** and their relative levels of abundance at FD, SD, and SS stages **(B)**. Different letters (a, b, and c) indicate significant differences among treatments according to Dunn’s test (*p* < 0.05), while the same letter signifies no significant difference.

We used random forest analysis to identify the endophytic fungal taxa distinguishing seeds at different stages (Figure 3A). Ranked by their importance value, the top 20 fungal OTUs mainly belonged to *Ascomycota*, specifically *Stachybotryaceae*, *Claussenomyces*, *Nectriaceae*, *Sordariales*, *Fusarium*, and *Trichoderma*. The relative abundance results showed that the four pathogenic fungi: *Stachybotryaceae*, *Claussenomyces*, *Sordariales*, and *Fusarium*, could be viewed as key taxa in the SS stage with a corresponding significant increase in the biocontrol fungus *Trichoderma*. The pathogen *Nectriaceae* could be considered a key taxon in the FS stage, showing a significantly higher abundance compared to the other two stages (Figure 3B).

### 3.3 Phenotypic, physiological characteristics and hormone content of PPC

We observed significant changes in the phenotype of PPC treated with sterile water, MT, SL, and BR (Table 1). In general, compared to CK, plants treated with MT depicted significantly enhanced robustness, with respective increases of 39.45, 10.34, 67.11, and 42.48% in root length, shoot length, fresh weight, and biomass (*p < 0.05*). SL did not affect the primary root growth significantly, but it led to a significant increase in shoot length, fresh weight, and biomass. BR notably improved shoot growth and exerted a distinct inhibitory effect on primary root growth, and did not significantly influence biomass accumulation. The activities of SOD and CAT were 32.81–108.48% and 20.63–42.86% higher in the three hormone-treated groups than the control, respectively (Table 1). Plants treated with MT exhibited low levels of MDA and H_2_O_2_. In contrast, SL and BR treatments significantly raised H_2_O_2_ levels, suggesting that the plants under these treatments might be experiencing oxidative stress. MT, SL and BR improved the Pn by10.74–40.00% than CK. Gs and Tr were 14.08–49.32% and 12.88–15.02% higher in MT and SL treatments, than CK, respectively, while Ci was significantly reduced. Treatment with BR significantly increased the gs and Ci transpiration rates compared to CK, while TR significantly decreased. With respect to the control, the phytohormone contents in the MT, SL, and BR treatments were 0.51, 0.69, and 0.34 mg/kg, respectively, marking an increase of 66.66, 56.25, and 66.52%, respectively. The content of SA significantly increased under the influence of all phytohormone treatments.

**TABLE 1:** Phenotype, physiological properties and phytohormone content of *Paris polyphylla var. chinensis (Franch.) Hara* (PPC) treated with different phytohormones.

Plant index	Treatments			
	**CK**	**MT**	**SL**	**BR**
Root length (cm)	3.63 ± 0.15b	5.07 ± 0.15a	3.47 ± 0.06bc	3.3 ± 0.1c
Shoot length (cm)	0.97 ± 0.06c	1.07 ± 0.06b	1.21 ± 0.02a	1.07 ± 0.02b
Fresh weight (g)	7.50 ± 0.20c	12.53 ± 0.40a	9.50 ± 0.10b	7.23 ± 0.25c
Biomass (g)	4.40 ± 0.10c	7.45 ± 0.15a	5.07 ± 0.40b	4.87 ± 0.25bc
SOD activity (U/g)	20.05 ± 1.51d	31.96 ± 0.79b	41.80 ± 2.54a	26.63 ± 1.19c
CAT activity (U/g)	854.28 ± 48.89c	1211.36 ± 10.36a	1030.56 ± 52.95b	1220.4 ± 17.94a
H_2_O_2_ content (μmol/g)	473.86 ± 4.25c	421.75 ± 19.78c	612.28 ± 1.32a	539.12 ± 58.68b
MDA content (nmol/g)	66.56 ± 2.68a	32.25 ± 0.93d	55.38 ± 1.42b	38.36 ± 4.73c
Pn (μmol⋅CO_2_⋅m^–2^⋅s^–1^)	3.92 ± 0.32c	5.49 ± 0.24a	4.62 ± 0.37b	4.35 ± 0.31bc
Gs (mmol⋅CO_2_⋅m^–2^⋅s^–1^)	0.031 ± 0.00c	0.03 ± 0.00b	0.05 ± 0.00a	0.05 ± 0.00a
Ci (μmol CO_2_ mol^–1^)	48.96 ± 2.85b	36.86 ± 0.95d	44.75 ± 3.44c	53.16 ± 2.06a
Tr (mmol⋅H_2_O⋅m^–2^⋅s^–1^)	1.96 ± 0.10b	2.21 ± 0.02a	2.26 ± 0.04a	1.78 ± 0.08c
Salicylic acid content (mg/kg)	15.39 ± 0.31c	26.16 ± 0.89a	19.30 ± 3.52b	22.83 ± 0.37a
Melatonin content (mg/kg)	0.31 ± 0.05b	0.51 ± 0.06a	0.21 ± 0.03b	0.21 ± 0.06b
Strigolactone content (mg/kg)	0.44 ± 0.04b	0.49 ± 0.03b	0.69 ± 0.14a	0.42 ± 0.03b
24-epibrassinolide content (mg/kg)	0.21 ± 0.02c	0.24 ± 0.01bc	0.29 ± 0.04ab	0.34 ± 0.03a

Data are the means of three replicates with standard deviations (±SD). Different letters (a, b, c, and d) indicate significant differences among treatments according to Dunn’s test (*p* < 0.05), while the same letter signifies no significant difference. CK, sterile water; MT, Melatonin; SL, strigolactone; BR, 24-epibrassinolide.

### 3.4 Variations in the diversity of rhizosphere soil microbial communities of PPC following phytohormone treatments

**FIGURE 4:**
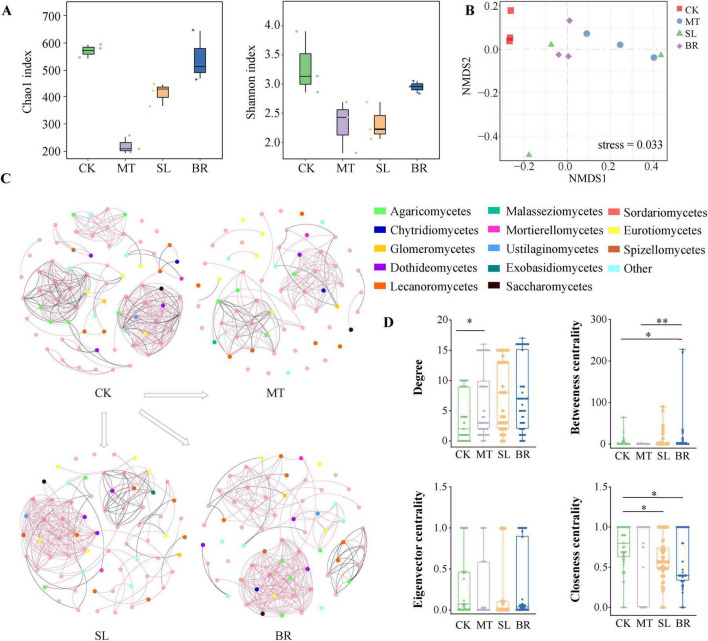
Endophytic fungal diversity and community composition of *Paris polyphylla var. chinensis (Franch.) Hara* (PPC) in different phytohormone treatments. **(A)** Alpha diversity data (Chao1 and Shannon index). **(B)** Non-metric multidimensional scaling (NMDS). **(C)** Co-occurrence networks at class level based on a correlation analysis. A link represents a significant correlation (Spearman’s |r| > 0.85 and FDR-corrected *p* < 0.05). Red and black links represent positive and negative relationships, respectively. **(D**) Comparison of node-level topological characteristics at class level. *p < 0.05, **p < 0.01 (Dunn’s test).

We found that the Chao1 and Shannon indices of endophytic fungi in PPC under MT and SL treatments were significantly lower in comparison to CK (Figure 4A). We further analyzed the variations in soil microbial community structure under phytohormone treatments using NMDS analysis (Figure 4B). The NMDS plot revealed a statistically significant separation (*p* < 0.05) between the microbial communities of the CK treatment and those associated with MT, SL, and BR treatments, indicating that phytohormone application significantly altered microbial community composition.

At the class level, *Sordariomycetes*, *Eurotiomycetes*, *Mortierellomycetes*, and *Leotiomycetes* were dominant in all samples (Figure 4C). Based on pertinent findings, we derived the co-occurrence network of the PPC endophytic fungal community across CK and various hormone treatments (Figure 4C). CK had 290 edges and 76 nodes, MT had 128 edges and 65 nodes, SL had 210 edges and 68 nodes, and BR had 240 edges and 67 nodes. MT demonstrated the fewest nodes and edges. We studied unique community-level topological traits at the node level, chiefly focusing on degree, betweenness centrality, closeness centrality, and eigenvector centrality (Figure 4D). The degree, betweenness centrality, and eigenvector centrality of BR were notably higher than those of CK, suggesting a more pronounced level of symbiotic connection.

### 3.5 Potential effects of phytohormone, antioxidant system, photosynthetic rate, and fungal communities on PPC biomass

**FIGURE 5:**
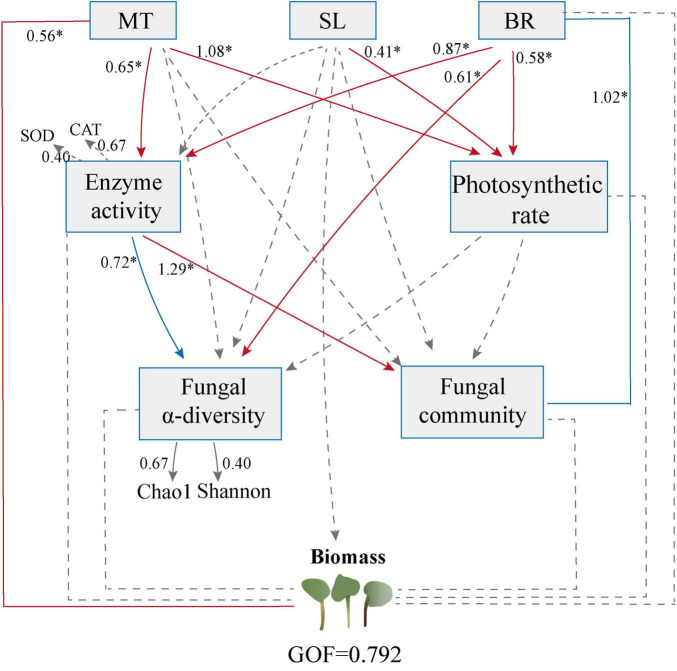
Partial least squares-path modeling (PLS-PM) analysis of direct and indirect influences of phytohormone contents, physiological properties and endophytic fungal communities on *Paris polyphylla var. chinensis (Franch.) Hara* (PPC) biomass. Positive and negative effects are presented by red and blue arrows, respectively. Path coefficients that were insignificantly different from zero are shown as dashed lines; *p < 0.05. The goodness-of-fit was used to assess the model.

To examine the potential direct and indirect effects of phytohormone, antioxidant systems, photosynthetic rate, and fungal communities on PPC biomass, we conducted a PLS-PM analysis based on the effects and relationships of known predictors (Figure 5). The final model fit our dataset well and suggested that the MT content could be the most significant factor directly influencing the PPC biomass. MT, SL, and BR appeared to directly influence the photosynthetic rate. Additionally, MT and BR might modulate the diversity of endophytic fungi indirectly through their influence on antioxidant enzyme activities. Furthermore, BR exerts a direct effect on the diversity of endophytic fungi. Interestingly, neither the α-diversity of endophytic fungi (including Chao1 and Shannon) nor the structure of their community (NMDS1) yielded a direct and significant influence on biomass.

### 3.6 Discrimination of PPC endophytic fungi at different phytohormone treatments

**FIGURE 6:**
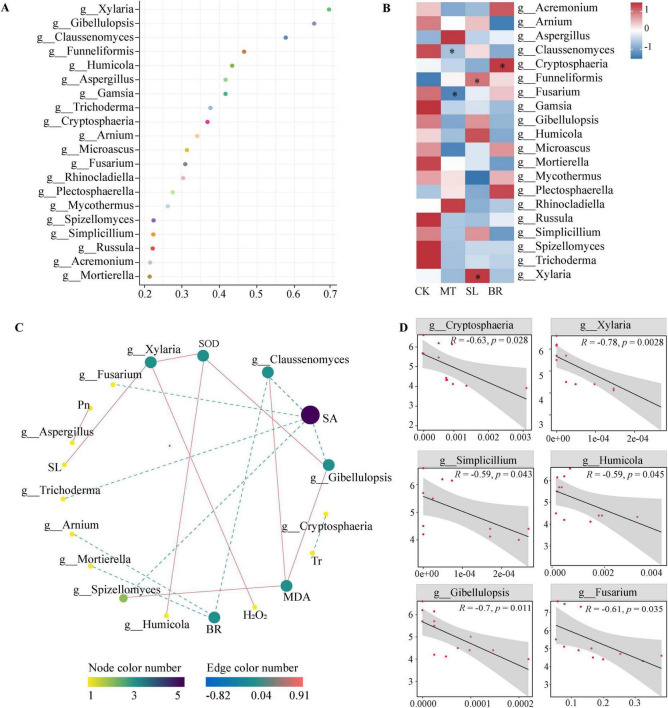
Potential biomarkers of endophytic fungi in response to different phytohormone treatments and their relative abundance in relation to plant physiology and biomass. **(A)** Genus annotation of the top 20 endophytic fungi in random forest model. **(B)** The heatmaps exhibit the changes in the relative abundance in response to different phytohormone treatments. *p < 0.05 indicate significant differences the treatments compared to CK (Dunn’s test). **(C)** Pearson analysis of phytohormone contents, physiological properties and biomarkers. A link represents a significant correlation (Pearson’s |r| > 0.5, and FDR-corrected *p* < 0.05). Red and blue links represent positive and negative relationships, respectively. **(D**) Linear regressions between the relative abundances of biomarkers and the *Paris polyphylla var. chinensis (Franch.) Hara* (PPC) biomass. The lines represent the least squares regression fit, and the shaded area are used to indicate the 95% confidence intervals. CK, sterile water; MT, Melatonin; SL, strigolactone; BR, 24-epibrassinolide.

We applied the random forest model to regress the relative abundance of root endophytic fungi at the genus level in PPC against different phytohormone treatments, determining which endophytic fungal taxa set apart from the different hormone treatments (Figure 6A). The model depicted the top 20 root microbiota with a variation index associated with different hormones. Figure 6B illustrates the specific shifts in significant fungal taxa under different hormone treatments, with MT augmenting the abundance of *Funneliformis*. SL notably increased the abundance of *Funneliformis* and significantly intensified the abundance of the pathogenic fungus *Xylaria*. BR markedly increased the abundance of *Cryptosphaeria*. A linear regression analysis revealed that among the key genera, *Cryptosphaeria* and *Xylaria* significantly negatively influenced biomass.

Pearson correlation analysis showed that indicated that endophytic fungi had a stronger association with phytohormone and antioxidant systems than with photosynthetic systems (Figure 6C). The abundances of fungal genera *Arnium*, *Claussenomyces*, and *Mortierella* were found to negatively correlate with PPC BR content. The abundance of genera *Spizellomyces*, *Claussenomyces*, and *Gibellulopsis* showed a positive correlation with plant MDA content. SL content was found to have a significant negative correlation with *Xylaria*. Furthermore, there was a significant positive correlation between Pn and *Aspergillu*s, as well as a significant negative correlation between Tr and *Cryptosphaeria*.

Owing to the notable changes within the endophytic fungal community in PPC spurred by phytohormones, we delved deeper into their potentially interlinked consequences on the increase in PPC biomass (Figure 6D). Our findings point out that among the key taxa significantly influencing alterations in the endophytic community under specified treatments with various phytohormones, species notably impacting biomass include *Cryptosphaeria* (R = −0.63, *p* = 0.028), *Xylaria* (R = −0.78, *p* = 0.002), *Simplicillium* (R = −0.59, *p* = 0.043), *Humicola* (R = −0.59, *p* = 0.045), *Gibellulopsis* (R = −0.7, *p* = 0.011), and *Fusarium* (R = −0.61, *p* = 0.035). Each of these revealed significant negative correlations with PPC biomass.

## 4 Discussion

### 4.1 Endophytic fungal dynamics in fresh seeds, sand-stored seeds, and seedlings

The study results indicate that there is no significant change in endophytic fungal diversity after PPC seeds are stored in sand. This can be attributed to the protective function of the seed coat, the outermost layer of PPC seeds, which shields the seeds from mechanical damage and prevents the invasion of diseases and pests (Zhang et al., 2023). Notably, *Fusarium* and *Plectosphaerella* are significantly enriched as potential biomarkers during this stage, with reports suggesting that these fungi are reputed for causing crop root rot and wilt through widespread colonization (Franke-Whittle et al., 2015; Yang et al., 2023).

*Paris polyphylla var. chinensis (Franch.) Hara* seedlings significantly alter the diversity and community structure of their endophytic fungi. It is posited that this shift is associated with the shedding of the seed coat, which initiates endophytic fungal interaction with the surrounding environmental microbiota. In this study, the increased relative abundance of symbiotic microorganisms such as *Aspergillaceae*, *Coniochaetales*, and *Trichoderma* during seed germination is ascribed to the efficient nutrient supply from the endosperm formed during germination, fostering the growth of the microbial communities within the seed. These microbial communities, possibly in a dormant state inside the seed, utilize the nutrients released during germination to break dormancy (Barret et al., 2015; Torres-Cortés et al., 2018).

The phylum *Ascomycota* was the most abundantly annotated group of endophytic fungi at various stages of PPC seed germination. In our study, the early seedling growth stage led to the enrichment of multiple *Ascomycota* biomarkers, including *Gibellulopsis* and *Cryptosphaeria*, two potential plant pathogens that may detriment growth (Trouillas and Gubler, 2016; Liao et al., 2024), as well as *Sordariomycetes*, a prevalent category with anti-pathogenic activities in plants (Wang et al., 2023). Furthermore, we found that the topological values of the *Sordariomycetes* taxa were significantly higher than those of other groups, indicating that *Sordariomycetes* often occupy central positions in the network. Therefore, we presuppose that *Sordariomycetes* plays a significant role during the PPC seedling growth stage.

### 4.2 Responses of plant phenotype and physiology to phytohormones addition

The pot experiment demonstrated that both MT and SL significantly promoted PPC biomass accumulation. On the one hand, it has been shown that three phytohormones enhanced the Pn rate to varying degrees, providing plants with more carbon sources for synthesis and positively affecting plant growth (Yan D. et al., 2023). On the other hand, all three phytohormones can regulate the production and elimination of reactive oxygen, which in turn affects various aspects of plant growth and development (Devireddy et al., 2021). The three hormones notably increased the levels of SOD and CAT. MT exhibited lower levels of MDA and H_2_O_2_, implying that the oxidation-reduction state of the plant is relatively stable. Numerous reports have suggested that MT primarily functions to protect plants from various abiotic and biotic stresses by scavenging ROS and regulating antioxidant levels (Tan et al., 2012). Moreover, studies have found that MT does not significantly impact the membrane lipid peroxidation system (MDA and H_2_O_2_) of the plant under normal growth conditions but can decrease their MDA and H_2_O_2_ content under oxidative stress conditions (He et al., 2023; Darré et al., 2024). Based on this evidence, we posit that the natural slow growth of PPC seedlings may be due to oxidative stress in their environment. Therefore, additional supplementation of phytohormones for scavenging reactive oxygen species is deemed necessary. The inhibition of root growth in the BR treatment may be due to the relatively high concentration chosen for this study, which may not be optimal for PPC plants (Lv et al., 2018). Under SL treatment, a significant increase in H_2_O_2_ content and root length was observed, while shoot growth declined. The study revealed that the SL-induced accumulation of H_2_O_2_ initiates various reactions such as transcription and oxidative modification, ultimately stimulating root development (Tian et al., 2018). The restriction of shoot growth aligns with the conclusions drawn by Chen et al. (2009). Encouragingly, MT exerted a stronger effect than SL and BR on nearly all growth parameters. To some extent, this supports the role of melatonin as a primary regulator in PPC, in agreement with previous research findings (Carvalhais et al., 2019).

### 4.3 Responses of plant endophytic fungal microbial communities and key taxa to phytohormones addition

Manipulating the endophytic microbial communities in natural environments is critical for leveraging ecological interactions to enhance plant productivity. Therefore, it is essential to conduct pot experiments in uncontrolled, natural conditions, to examine the impact of phytohormones on the endophytic fungal community of PPC. In this study, all three phytohormones significantly altered the structure of the endophytic fungal community. We used PLS-PM to assess the relationships between hormone concentrations, physiological parameters (such as antioxidant enzyme activities and photosynthetic rates), endophytic fungal community composition (diversity and structure), and plant biomass. In the current study, MT and BR indirectly affected endophytic fungal diversity by enhancing the activity of antioxidant enzymes. Fungi like *Gibellulopsis*, *Spizellomyces*, and *Claussenomyce*s showed significant positive correlations with MDA content, possibly due to the hypersensitive response triggered by plant infection and fungal recognition, which leads to increased lipid peroxidation of the cell membrane (Berrios and Rentsch, 2022). SOD and CAT are vital protective enzymes involved in scavenging ROS in plants. The coordination of ROS levels with the abundance and diversity of microbial populations is well-documented (Fuller et al., 2017). *Xylaria* and *Humicola* demonstrated significant positive correlations with SOD activity, which may be due to the tendency of commensal fungal genomes to enrich genes for ROS-scavenging enzymes (Berrios and Ely, 2020). MT content had a direct positive effect on biomass, but the diversity and overall structure of the endophytic fungal community did not vastly influence biomass. We conducted a subsequent random forest analysis to identify potential biomarkers of the endophytic fungal community responding to various phytohormone treatments. These key taxa are generally considered critical factors in differentiating microbial community structure and they exert varied effects on plant growth. In this study, MT exerted a significant positive impact on the biomass of PPC by markedly inhibiting the abundance of the pathogenic fungus *Fusarium*. The study showed the significant enrichment in two key taxa, *Funneliformis* and *Xylaria*, in that SL. *Xylaria* is identified as a pathogen that negatively influences crop root development (Garcia-Aroca et al., 2021). The results of linear analysis also indicate a negative correlation between *Xylaria* and biomass. In the BR treatment, *Cryptosphaeria* was significantly enriched, and reports have demonstrated that this pathogen can cause cankers in crops and forest plants (Yan C. et al., 2023). Consequently, *Cryptosphaeria* could be a key taxon responsible for the adverse effects of BR on PPC growth. In conclusion, the effects of various plant hormones on the endophytic fungi within PPC are specific and complex. MT, SL, and BR can regulate the antioxidant system, thereby influencing diversity and structural alterations within the endophytic fungal community. The findings of this study underline the view that phytohormones indirectly impact microbial communities by modifying plant physiological properties (Eichmann et al., 2021; Shao et al., 2024). The effects of MT, SL, and BR on the biomass of PPC are independent of changes in microbial community structure. Nevertheless, it is connected to the succession of specific key biomarker species affected by each hormone.

## 5 Conclusion

In summary, the study of the endophytic fungal microbiome in FS, SSD, and SS stages of PPC indicates that the seedling stage exhibits the greatest microbial richness and diversity, along with a significant shift in composition. We also identified *Sordariomycetes* as essential microorganisms in this process, potentially contributing to the establishment and growth of PPC seedlings. Moreover, this study explored the effects of phytohormones on the phenotypic, physiological characteristics, and endophytic fungal community of PPC. Our findings showed that MT and SL significantly boosted PPC biomass by 69.32 and 15.23%, respectively. However, 2 mg/L of BR hindered PPC root development. MT, SL, and BR notably induce changes in the endophytic fungal community composition and diversity within PPC, potentially affecting biomass through the specific regulation of potential biomarkers. Moreover, phytohormones can indirectly impact the endophytic fungal community by updating the plant antioxidant system. This research provides novel insights into the dynamics of microbial communities within FS, SSD, and SS. The variations among distinct phytohormones may ultimately enhance our predictive understanding regarding strategies to control plant microbiota, either directly or indirectly, for improving plant health.

## Data Availability

The datasets presented in this study can be found in online repositories. The names of the repository/repositories and accession number(s) can be found below: https://www.ncbi.nlm.nih.gov/, PRJNA1193989.

## Footnotes

1
https://unite.ut
